# Case report and review of literature: Isolated intramedullary spinal neurocysticercosis

**DOI:** 10.3389/fneur.2022.1030468

**Published:** 2022-11-10

**Authors:** Diana Andino, John T. Tsiang, Nathan C. Pecoraro, Ronak Jani, Jordan C. Iordanou, Jehad Zakaria, Ewa Borys, David D. Pasquale, Russ P. Nockels, Michael J. Schneck

**Affiliations:** ^1^Department of Neurology, Loyola University Medical Center, Maywood, IL, United States; ^2^Department of Neurological Surgery, Loyola University Medical Center, Maywood, IL, United States; ^3^Department of Neurological Surgery, Riverside Medical Center, Kankakee, IL, United States; ^4^Department of Pathology and Laboratory Medicine, Loyola University Medical Center, Maywood, IL, United States; ^5^Department of Radiology, Loyola University Medical Center, Maywood, IL, United States

**Keywords:** neurocysticercosis, spine, lesion, cyst, case report, resection, intramedullary

## Abstract

**Background:**

Cases of isolated intramedullary spinal neurocysticercosis are extremely rare. Only 25 cases have been reported before 2022. Due to its rarity, the diagnosis of spinal neurocysticercosis may be missed.

**Case presentation:**

We describe a 37-year-old female patient who developed back pain and lower extremity weakness and was found to have an intramedullary thoracic spine cystic lesion. She was taken to the operating room for resection of the lesion. Pathology revealed a larval cyst wall consistent with neurocysticercosis. The patient was started on albendazole and dexamethasone. Her exam improved post-operatively, and she was able to ambulate with minimal difficulty at the time of follow up.

**Conclusion:**

The case provides insights on the diagnosis and treatment of isolated intramedullary spinal neurocysticercosis. Review of the literature suggests that combined surgical and medical intervention results in significant improvement in the patient's neurological exam, and decreases morbidity associated with the disease. We propose a treatment paradigm for this rare manifestation of neurocysticercosis.

## Introduction

Cysticercosis is the most common parasitic disease worldwide that affects the nervous system (1). It is caused by the cestode *Taenia solium* with pigs serving as an intermediate host. When humans ingest larvae in undercooked pork, the infection is limited to gastrointestinal tract, where mature worms develop. In neurocysticercosis, humans become intermediate hosts by ingesting the ova, not the larvae, usually from fecal contamination of water and contaminated vegetables, and develop disseminated disease (2). Cysts that form within the nervous system are primarily found in the brain, with only an estimated 1% of cases with spinal cysts (3). Isolated intramedullary spinal neurocysticercosis is extremely rare, however, with few cases reported in literature before 2022. Given the non-specific presenting symptoms and increasing incidence, intramedullary spinal neurocysticercosis should be considered during the evaluation of suspicious cases with appropriate imaging. Here, the authors present a case of isolated intramedullary spinal neurocysticercosis and discuss the diagnostic evaluation, intervention, and current literature behind this disease process.

## Methods

A PubMed search was performed for articles indexed through the Medline database. Keywords used included “spinal” AND “neurocysticercosis” AND “isolated.” Fifty articles were identified, of which 16 were case reports of interest. Twenty-five patients were described within these 16 case reports published between 1993 and 2022 (Table 1). Thirty-four articles were excluded: fifteen articles were not about isolated intramedullary neurocysticercosis, eleven were not case reports, four were not isolated spinal cases, three articles were inaccessible and without abstract, and one article was not related to neurocysticercosis. A qualitative analysis was performed on the included cases, and findings are presented.

**Table 1:** Literature review on cases of isolated intramedullary spinal neurocysticercosis.

**References**	**PMID**	**Age**	**Location of lesion**	**Number of cysts**	**Clinical signs**	**Treatment**	**Resolution of symptoms**
Yang et al. (4)	35193508	23	Thoracic	Single	Motor, sensory	Surgery	Yes
		24	Thoracic	Single	Back pain, motor	Surgery	Yes
		47	Thoracic/Lumbar	Multiple	Bowel/bladder, motor, sensory	Surgery, Albendazole, Decadron	Yes
		27	Lumbar/Sacral	Single	Back pain, motor	Surgery	Yes
		38	Thoracic	Single	Motor, sensory	Surgery	Yes
		35	Thoracic	Single	Motor, sensory	Surgery	Yes
Dhar et al., (5)	34926816	35	Lumbar	Multiple	Back pain, sensory	Surgery, Albendazole, Decadron	Yes
Vadher et al. (6)	34113502	20	Thoracic	Multiple	Motor, sensory	Surgery, Albendazole, Decadron	Yes, but not complete
Jobanputra et al. (7)	32123621	44	Cervical	Single	Sensory	Surgery	Yes
Maste et al. (8)	29492150	30	Thoracic	Single	Back pain, motor, sensory	Albendazole, decadron	Yes
Datta et al. (9)	28602886	70	Thoracic	Single	Motor, sensory	Albendazole, decadron	Yes
		23	Thoracic	Multiple	Motor, sensory	Surgery, Albendazole, Decadron	Yes, but not complete
		24	Thoracic	Single	Motor, sensory	None	Yes, but not complete
Salazar et al. (10)	25595849	43	Cervical/Thoracic	Single	Motor, sensory	Surgery, Albendazole, Decadron	Yes
Qazi et al., (11)	25540546	19	Thoracic/Lumbar	Single	Bowel/bladder, motor, sensory	Surgery	Yes
Agale et al. (12)	22870160	38	Thoracic	Single	Motor	Surgery, Albendazole	Yes, but not complete
Azfar et al. (13)	21977090	10	Thoracic	Single	Bowel/bladder, motor, sensory	Albendazole, decadron	Yes
Lambertucci et al. (14)	22146927	23	Cervical	Single	Back pain, motor	Surgery, Albendazole, Decadron	Unknown
Vij et al. (15)	22234147	25	Thoracic	Single	Back pain, bowel/bladder, motor, sensory	Surgery, Decadron	Unknown
Gonçalves et al. (16)	20147871	62	Thoracic	Single	Yes, but not defined	Surgery	Yes
Bouree et al. (17)	17153691	20	Thoracic	Single	Back pain, motor	Surgery	Yes, but not complete
Colli et al. (18)	15926788	15	Thoracic	Not stated	Yes, but not defined	Surgery	No
		24	Lumbar/Sacral	Not stated	Sensory	Surgery	Yes, but not complete
		51	Cervical	Not stated	Yes, but not defined	Surgery	No
Sheehan et al. (19)	15926780	16	Cervical	Single	Sensory	Surgery, Praziquantel, Decadron	Yes, but not complete

## Case report/case presentation

A 37-year-old woman with no other medical history presented for evaluation of bilateral lower extremity weakness. She had been experiencing back pain for 3-weeks prior to presentation and was evaluated at an emergency department with initial onset of low back pain; she obtained a roentgenogram of her low back and was then discharged with a diagnosis of muscle strain. Three days after her initial evaluation, the patient began having lower extremity weakness, and presented for re-evaluation a week later; her weakness had progressed significantly. Patient was transferred from an outside emergency department to our institution. Her examination revealed bilateral lower extremity weakness, loss of sensation to the level of her umbilicus, urinary retention and constipation. When queried, she denied recent international travel: she had immigrated from Guatemala 15 years prior and has not returned since. She additionally denied ingestion of undercooked meats and being near livestock. She also denied headaches, seizures, or recent weight loss.

**Figure 1:**
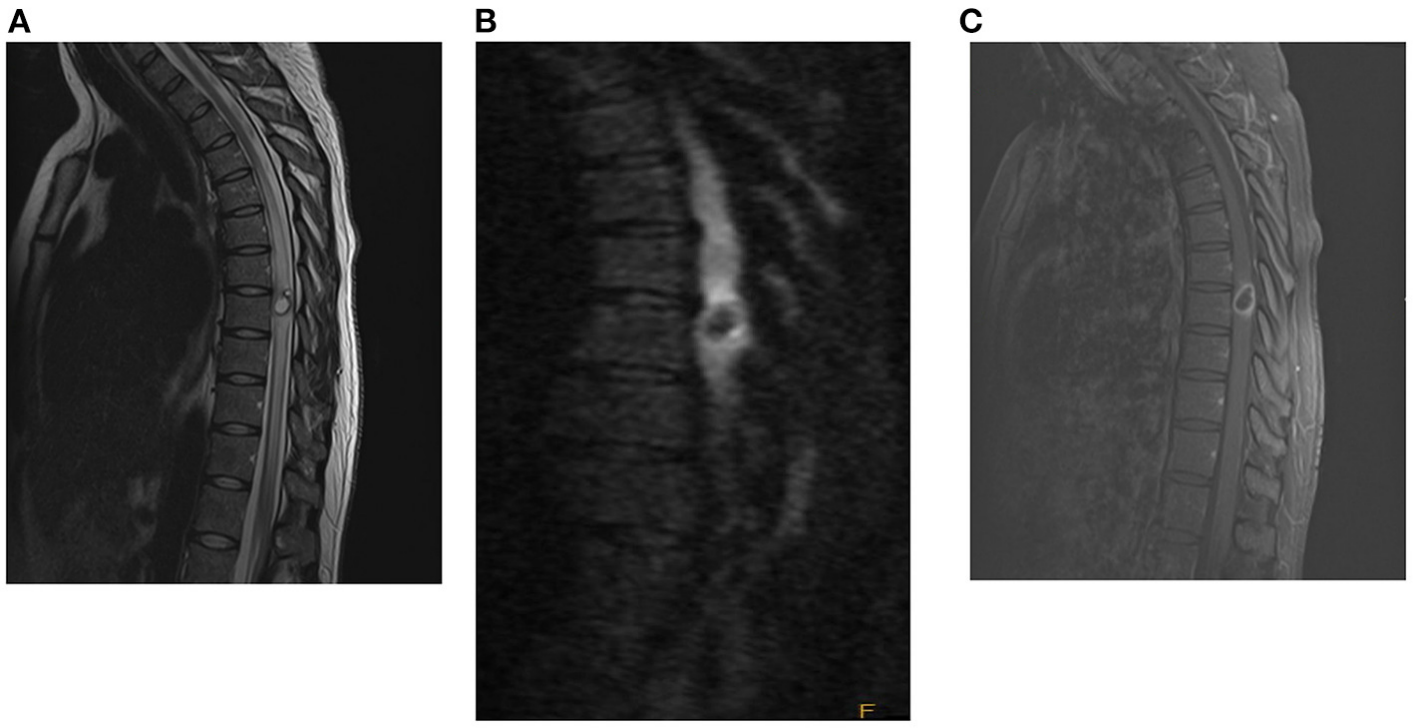
Pre-operative MRI with and without contrast of spine [**(A)**: T2, **(B)**: T1 wo contrast, **(C)**: T1 w contrast], demonstrating a rim-enhancing cystic lesion in the mid-thoracic spine with diffuse surrounding cord edema.

A magnetic resonance imaging (MRI) scan of her brain and entire spine demonstrated a ring-enhancing lesion at T8 with significant surrounding edema measuring 1.4 x 1.0 cm (shown in Figure 1). A small coma-shaped area of restricted diffusion is present eccentrically within the cyst. At this time, there was concern that this lesion represented an autoimmune demyelinating lesion (multiple sclerosis vs. transverse myelitis), and the patient was started on corticosteroids with moderate improvement in her strength over the course of 2 days. Despite a negative cystericus serum antibody test, there was growing concern that steroids were treating symptoms and not the underlying pathology, and the patient was therefore taken to the operating room for exploration and resection of the lesion.

**Figure 2:**
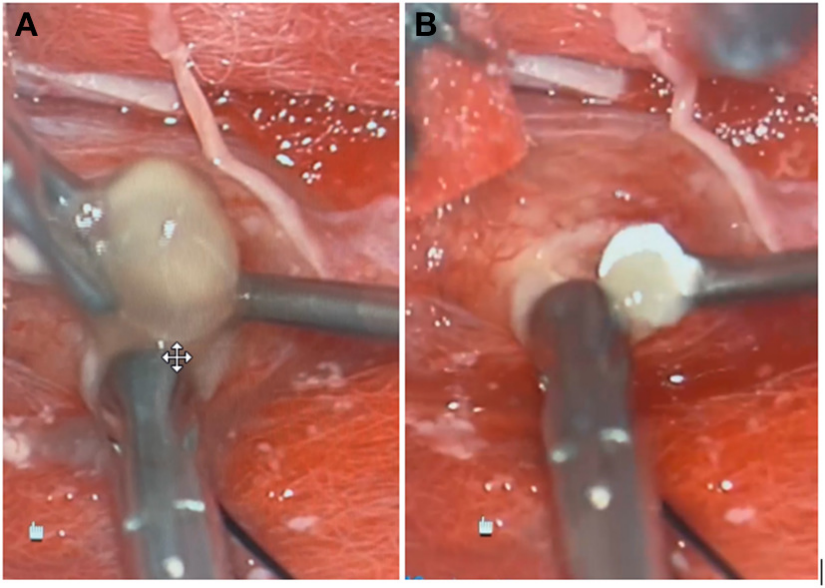
Gross specimen of lesions extracted during surgical intervention, one large **(A)** and one small **(B)**.

**Figure 3:**
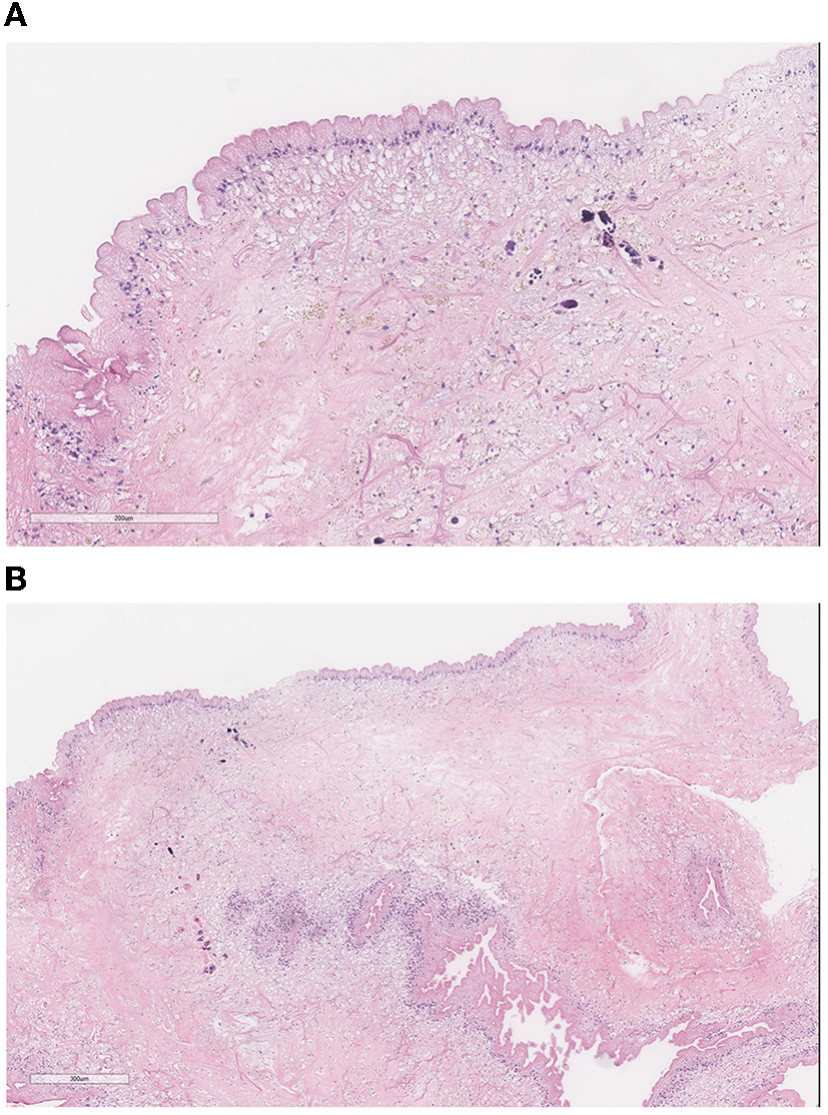
**(A)** Pathology demonstrates the characteristic three-layered wall of a **(B)** neurocysticercosis cyst: the undulating outer eosinophilic cuticular layer, the underlying cellular layer containing uniform small dark nuclei, and the inner reticular layer containing loosely arranged fibrils. Small amounts of calcification seen are consistent with the chronic nature of the infection.

In the operating room, a midline incision was made. Laminectomies were carried out from T7-T9 until the dura was adequately exposed. A midline durotomy was completed with localization through intraoperative ultrasound. Under high-intensity intraoperative microscopy, the posterior aspect of the spinal cord was observed to be extremely distorted due to the underlying lesion. A sharp incision was performed at the median sulcus and carried distally until a firmer portion of the lesion was encountered. A cystic component was then entered; purulence was noted on gross examination and suctioned away (shown in Figure 2). Multiple cultures, as well as portions of the capsule, were sent for pathological examination in both frozen and permanent sections. The preliminary pathological report returned as neurocysticercosis (shown in Figure 3). After removal of the capsule and cyst, the spinal cord was noted to be relaxed and pulsatile. A duraplasty was then performed utilizing an artificial dural graft material. The overlying muscle, fascia, subcutaneous tissue, and skin were then sequentially closed in a water-tight manner. The patient was extubated without complication and was admitted to the intensive care unit. Video highlights of the procedure are available in Supplementary materials.

**Figure 4:**
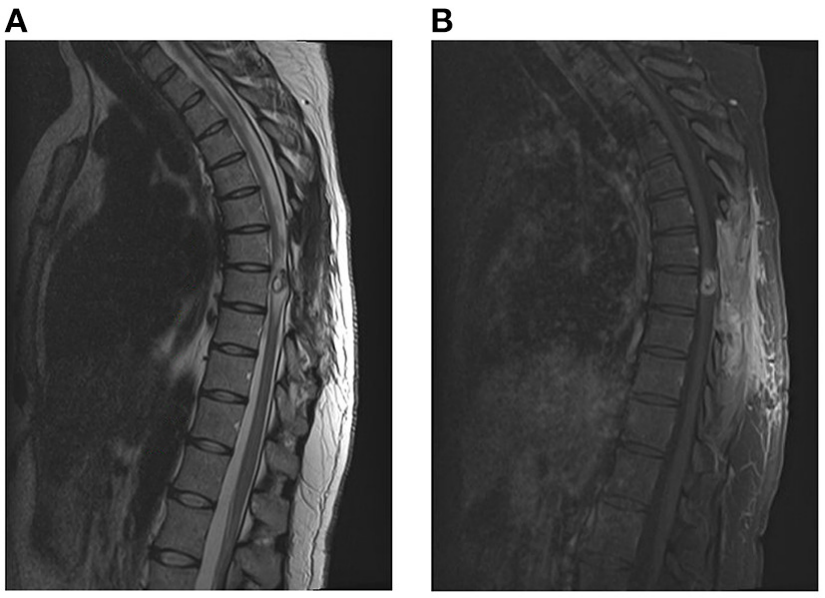
Post-operative MRI with and without contrast of the spine [**(A)**: T2, **(B)**: T1 w contrast], demonstrating interval decrease in size of the lesion as well as decrease in the edema surrounding the lesion.

In the following days, the patient was noted to have dramatic improvements in her bilateral lower extremity strength. She was started on albendazole (400 mg every 12 h) and dexamethasone (8 mg daily) for 14 days as per recommendations from Infectious Disease consultants. The remainder of her hospital course was uncomplicated. She was ultimately discharged to an Inpatient Rehabilitation Hospital and was noted to have full strength in her bilateral lower extremities and resolved urinary retention just prior to discharge, but remained with paresthesia in lower abdomen and lower extremities. She was then seen in the outpatient clinic 2 months later, where she stated that she had regained the majority of her strength in her lower extremities, and had significant improvements in her sensory complaints as well. An MRI was obtained demonstrating interval decrease in the size of lesion as well as decrease in the surrounding edema within the spinal cord (shown in Figure 4).

## Ethics

Informed consent was obtained from the individual for the publication of any potentially identifiable images or data included in this article.

## Discussion

Cysticercosis occurs when *Taenia solium* ova are ingested by the patient. The ova hatch in the intestine, and the larvae penetrate into the bloodstream and eventually lodge in host tissue. When the tissue involved is either the brain or the spine, the disease is called neurocysticercosis. While symptoms may develop as a result of mass effect from the cyst, cysts may also remain asymptomatic for many years (20). This may be the result of parasite-linked anti-inflammatory factors which inhibit both humoral and cellular immune responses to the cyst (21). When the cysticercus dies (either due to treatment or due to the parasite's lifespan), parasite-linked immunosuppression ceases and lesions can become symptomatic. This is likely the case with our patient, who did not have a scolex noted on surgical pathology indicating a live organism, but did have calcification suggestive of a long-standing lesion (22).

There are thus four recognized radiographic stages of a neurocysticercosis infection: (1) a vesicular stage with a parasite visible on imaging with little or no inflammation; (2) a colloidal vesicular stage where the parasite dies and there is increased surrounding edema and inflammation; (3) a granular nodular stage where the scolex is mineralized and the surrounding edema and inflammation decreases; and (4) a nodular calcified stage where the cyst completely mineralizes without surrounding inflammation (23).

Isolated intramedullary spinal neurocysticercosis is an extremely rare entity, and may diagnostically be difficult and unexpected, especially in areas of the world where *Taenia solium* is not endemic. *Taenia solium* is most prevalent in Latin America, Asia, and Africa; given global migration of persons, its incidence has been increasing in countries outside of its endemic region (24). Intracranial neurocysticercosis is the most common form, and spinal cases are rare. These cases account for 0.7–5.85% of all cases reported. Even rarer are isolated intramedullary lesions (11). Our case is illustrative of the diagnostic difficulty of intramedullary spinal neurocysticercosis. The patient had a precursory diagnosis of a demyelinating plaque. Only after re-review of MR imaging when the patient failed to improve did we reach the tentative diagnosis of neurocysticercosis that was subsequently confirmed on surgical pathology.

For diagnosis of spinal neurocysticercosis, a good clinical history is paramount (5). In our review of literature (Table 1), 17 cases (68%) exhibited symptoms of motor weakness, 16 (64%) were with sensory symptoms (either radiculopathy or numbness/tingling with sensory level), seven (28%) had low back pain, and three (12%) had bowel/bladder difficulties. Affected areas tend to be isolated towards the mid or distal spinal cord, as 4 patients (16%) had lesions in the cervical spine, 16 patients (64%) had lesions in the thoracic spine, and two patients (8%) had lesions in the upper lumbar spine. This is consistent with prior reports suggesting a predominance of lower thoracic lesions for intramedullary spinal neurocysticercosis (23). A history of pork consumption and/or travel to endemic areas (particularly areas without access to clean water) should also increase clinical suspicion for spinal neurocysticercosis; the cases we sampled did not mention these salient points with enough frequency for us to draw specific conclusions.

Given the non-specific symptoms, further clinical studies are needed to increase the diagnostic suspicion for neurocysticercosis. Laboratory studies can be used to test for antibodies to *Taenia solium*, but these have variable sensitivity and specificity and may be falsely negative in “light” infections (25). Imaging studies provide more diagnostic confidence (23). Characteristic neurocysticercosis lesions appear nodular or cystic on MRI. The scolex, if present, appears as a mural nodule isointense to the surrounding tissue on T1WI, iso- to hyperintense on T2WI, and hyperintense on DWI; there is additionally peripheral ring-like enhancement but no enhancement in the scolex (4). Neurocysticercosis lesions can have variable diffusion restriction depending on its stage, but typically will display minimal to no diffusion restriction. A small comma-shaped area of diffusion restriction, present in our patient, has also been described in literature as characteristic of a neurocysticercosis cyst. This contrasts with many other spinal lesions, including active demyelinating plaque, tumor, and bacterial abscesses, which typically will show restricted diffusion on DWI.

**Figure 5:**
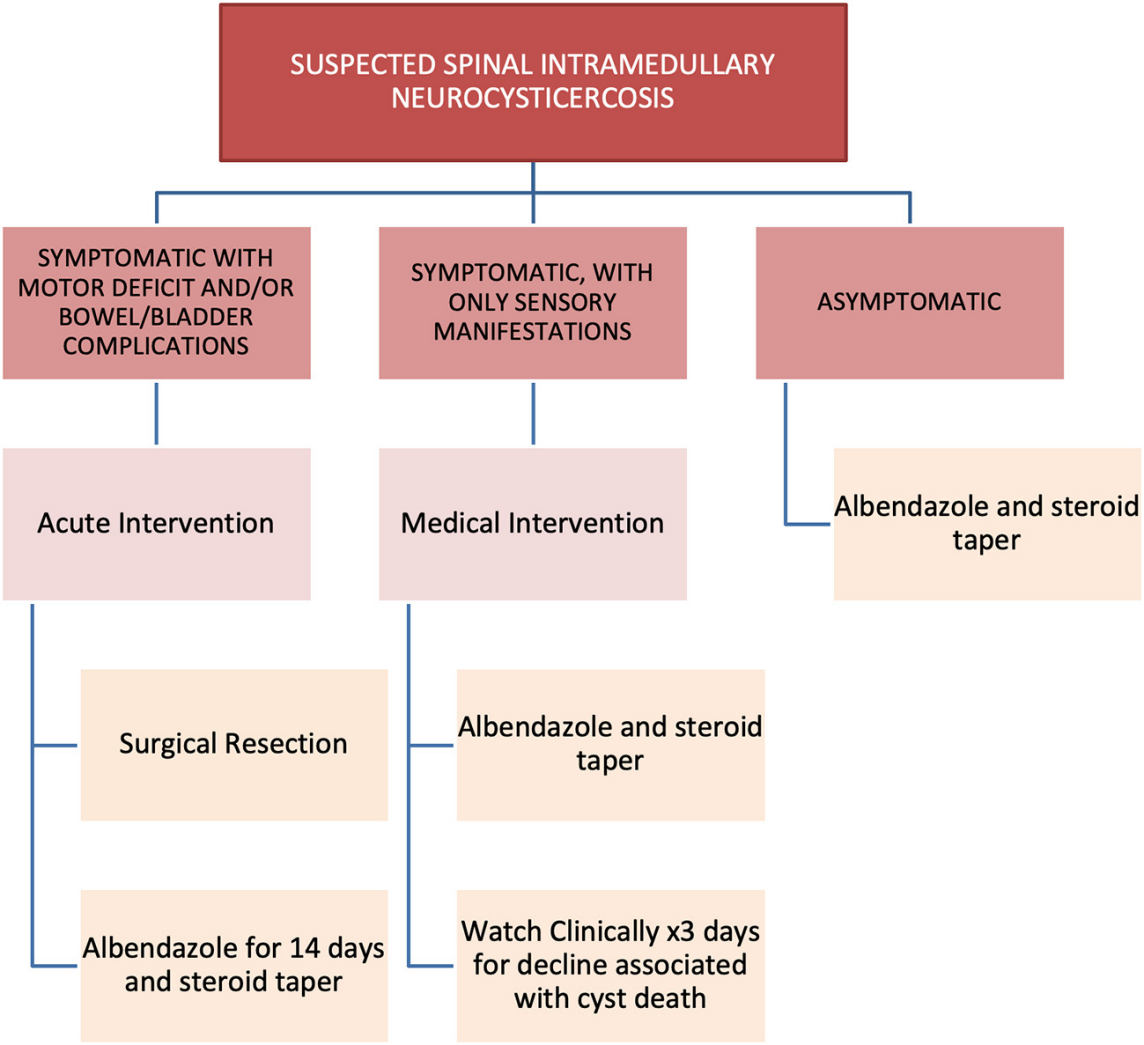
Proposed treatment protocol.

Due to the low incidence of isolated intramedullary spinal neurocysticercosis, treatment has not been standardized. In our literature review, 18 patients (82%) underwent surgical intervention to remove the spinal lesion. Piecemeal removal of cysticerci has not been shown to increase risk of disseminated disease. The treatment paradigm was not described for 3 patients. Ten patients (45%) received albendazole and dexamethasone; two patients (9%) received only dexamethasone. Fourteen patients (64%) experienced complete recovery; six (27%) experienced partial recovery; and no patients had worsening deficits. Two patients did not have their recovery described. Review of the data suggests that recovery was not related to modality of treatment, and more related to length of symptomatology. We therefore propose a treatment protocol for managing intramedullary spinal neurocysticercosis (shown in Figure 5).

This case illustrates the diagnostic difficulty of isolated intramedullary spinal neurocysticercosis. A thorough clinical history, exam, and associated clinical and radiographic studies can help to narrow the differential diagnosis. The interventions and treatments performed on this patient mirror those in the few documented cases in literature. Our treatment protocol therefore provides standardization and guidance in the treatment of this rare disease process.

## Data availability statement

The raw data supporting the conclusions of this article will be made available by the authors, without undue reservation.

## Ethics statement

Ethical review and approval was not required for the study on human participants in accordance with the local legislation and institutional requirements. The patients/participants provided their written informed consent to participate in this study. Written informed consent was obtained from the individual(s) for the publication of any potentially identifiable images or data included in this article.

## Author contributions

Conception and design: DA. Acquisition of data and drafting the article: DA and JT. Analysis and interpretation of data: DA, EB, DP, NP, and JT. Critically revising the article: DA, JT, NP, RJ, JI, JZ, EB, DP, RN, and MS. Reviewed final version of the manuscript and approved it for submission: EB, DP, RN, and MS.

## Conflict of interest

The authors declare that the research was conducted in the absence of any commercial or financial relationships that could be construed as a potential conflict of interest.

## Publisher's note

All claims expressed in this article are solely those of the authors and do not necessarily represent those of their affiliated organizations, or those of the publisher, the editors and the reviewers. Any product that may be evaluated in this article, or claim that may be made by its manufacturer, is not guaranteed or endorsed by the publisher.
